# Chronic consequences of ischemic stroke: Profiling brain injury and inflammation in a mouse model with reperfusion

**DOI:** 10.14814/phy2.16118

**Published:** 2024-06-23

**Authors:** Devin P. Murphy, Denali C. Dickson, Arisha N. Fatema, Nolan G. Carrasco, Kristian P. Doyle, Theodore P. Trouard, Helena W. Morrison

**Affiliations:** ^1^ Department of Biomedical Engineering, College of Engineering University of Arizona Tucson Arizona USA; ^2^ College of Nursing University of Arizona Tucson Arizona USA; ^3^ College of Pharmacy University of Arizona Tucson Arizona USA; ^4^ Department of Immunology, College of Medicine University of Arizona Tucson Arizona USA

**Keywords:** blood–brain barrier, chronic injury, foam cells, ischemic stroke

## Abstract

Stroke is a pervasive and debilitating global health concern, necessitating innovative therapeutic strategies, especially during recovery. While existing literature often focuses on acute interventions, our study addresses the uniqueness of brain tissue during wound healing, emphasizing the chronic phase following the commonly used middle cerebral artery (MCA) occlusion model. Using clinically relevant endpoints in male and female mice such as magnetic resonance imaging (MRI) and plasma neurofilament light (NFL) measurement, along with immunohistochemistry, we describe injury evolution. Our findings document significant alterations in edema, tissue remodeling, and gadolinium leakage through MRI. Plasma NFL concentration remained elevated at 30 days poststroke. Microglia responses are confined to the region adjacent to the injury, rather than continued widespread activation, and boron‐dipyrromethene (BODIPY) staining demonstrated the persistent presence of foam cells within the infarct. Additional immunohistochemistry highlighted sustained B and T lymphocyte presence in the poststroke brain. These observations underscore potentially pivotal roles played by chronic inflammation brought on by the lipid‐rich brain environment, and chronic blood–brain barrier dysfunction, in the development of secondary neurodegeneration. This study sheds light on the enduring consequences of ischemic stroke in the most used rodent stroke model and provides valuable insights for future research, clinical strategies, and therapeutic development.

## 1 INTRODUCTION

Stroke, a devastating brain injury, exacts a profound toll on both the United States (Tsao et al., 2023) and global populations (Thayabaranathan et al., 2022). However, the available therapeutic options are focused on reperfusion, achieved through either pharmacological or mechanical interventions (O'Collins et al., 2006). Recognizing the critical need for innovative treatments, the prevailing stroke literature places emphasis on investigating injury mechanisms and potential therapies during the acute phases (Banerjee & McCullough, 2022; O'Collins et al., 2006). Nevertheless, in recent years, a burgeoning body of research conducted in rodent stroke models has emerged, driven by the quest to decipher the mechanisms of brain healing during the chronic phases. This research aims to highlight the potential role of these processes in initiating or exacerbating poststroke complications, including the onset of dementia (Doyle & Buckwalter, 2017, 2020; Zbesko et al., 2023).

The brain's distinctive lipid‐rich composition distinguishes it from other organs. During subacute and chronic stages of stroke, the physical manifestation of the inflammatory response within the infarct bears a resemblance to atherosclerotic plaque (Adigun et al., 2023; Chung et al., 2018). This atherosclerosis‐like inflammatory response harbors an abundance of cholesterol‐laden macrophages/microglia, now transformed into dysfunctional foam cells due to overwhelmed lipid processing (Chung et al., 2018; Zbesko et al., 2023). These cells are inadequately separated from the surrounding brain tissue by permeable glial scars and can persist for an indeterminate duration, likely ranging from months to years (Sofroniew, 2009; Zbesko et al., 2018). This enduring consequence of stroke, along with the accompanying immune cells and inflammatory processes, has the potential to contribute to poststroke dementia and other chronic stroke complications, affecting approximately 30% of stroke patients (Becktel et al., 2022; Chung et al., 2018; Doyle & Buckwalter, 2017, 2020; Droś & Klimkowicz‐Mrowiec, 2021; Nguyen et al., 2016; Zbesko et al., 2018, 2023). The presence of the blood–brain barrier (BBB) also distinguishes the brain from other organs. The BBB is a complex and important structure that plays a vital role in protecting the brain. When the BBB is compromised, as can happen in stroke, harmful substances leak from the bloodstream into brain tissue (Morrison & Filosa, 2019). This also has the potential to contribute to poststroke dementia and other chronic stroke complications.

However, to date, a bulk of research exploring the chronic consequences of stroke has been conducted in male mice and has employed a stroke model that necessitates a craniectomy, resulting in a confined injury limited to a small cortical area and without incorporating reperfusion (Becktel et al., 2022; Chung et al., 2018; Doyle et al., 2015; Doyle & Buckwalter, 2017; Zbesko et al., 2018). This stands in contrast to the model more commonly used to investigate acute stroke that occludes the ostia of the MCA, producing a larger brain injury that spans multiple regions and includes the critical element of reperfusion. This distinction gains particular significance considering the recent successful development of thrombectomy procedures for the treatment of acute ischemic stroke. Consequently, the objective of this study was to describe brain injury, to emphasize the chronic phase, following the commonly used intraluminal filament model of middle cerebral artery occlusion (MCAO) with reperfusion in the brains of male and female mice. For our approach, we combined magnetic resonance imaging (MRI), functional activity assessments, assays for plasma neurofilament light (NFL) concentrations, and immunohistochemistry (IHC) to document BBB impairment, the chronic inflammatory response, and their relationship to recovery. With this comprehensive study, we hoped to shed light on the long‐term effects of ischemic stroke in the most widely used rodent stroke model and offer insightful information for future studies.

## 2 MATERIALS AND METHODS

### 2.1 Animals

Adult male and female mice (15 weeks, C57BL/6J) were purchased from Jackson Laboratories (Bar Harbor, ME). Animal handling and experimental procedures were performed with approval and in compliance with the University of Arizona Institutional Animal Care and Use Committee. The principles of laboratory animal care (NIH publication no. 86‐23, revised 1985) were followed, as well as specific national laws, where applicable. Mice were housed with a 12‐hr light/dark schedule (7 am–7 pm) with food and water available ad libitum (NIH‐31 diet, Inotive, IN). Experiments were carried out after at least 1 week of acclimation postarrival.

### 2.2 Study design and stroke model

**FIGURE 1:**
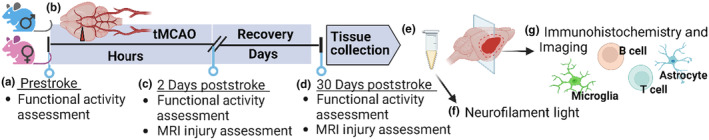
Study design and experiment timeline. Following a 5–7‐day acclimation period to their housing environment, mice underwent functional activity assessment (a), followed by a surgical procedure to induce transient middle cerebral artery occlusion (b). Following ischemic stroke, functional activity assessment, and magnetic resonance imaging (MRI) were carried out at 2 and 30 days poststroke (c and d) after which plasma and tissue (e) were collected at 30 days postinjury. Plasma was assessed for NFL concentration (f) and brain tissue (g) was processed for immunohistochemistry to detect microglia (IBA1) astrocytes (GFAP), B lymphocytes (B220), and T cells (CD3ε). BODIPY was used to assess lipid accumulation. Created with BioRender.com (YZ26KHM453).

The ARRIVE guidelines were used for transparent reporting of research methods and findings (Percie du Sert et al., 2020). The study design is summarized in Figure 1. A temporary ischemic stroke was induced in all mice using the filament method as previously described (Morrison & Filosa, 2013). Briefly, a filament [blunted 6–0 nylon suture (Ethilon, Ethicon) with a silicone coating (Xantopren cofort light, Heraus, New York) tip measuring 0.22–0.25 mm in diameter] was advanced to the MCA ostea via the internal common carotid artery to occlude the right MCA. When the right common carotid artery was tied, a two‐step decrease (first with carotid tie and second with correct filament placement) in relative cerebral blood flow was observed and confirmed via a laser Doppler (Preimed Periflux 5000, North Royalton, OH) placed periorbital and over the MCA territory. Ischemia continued for 45 min. To initiate reperfusion, the filament was removed, and the common carotid artery was untied. To be included in this study, animals must have experienced a decrease in relative cerebral blood flow of at least 70% of baseline (ischemia) accompanied by reperfusion, at least a 70% recovery of baseline cerebral blood flow values. All mice were anesthetized with 1–2% isoflurane in a 0.4 L/min medical air/0.1 L/min oxygen mixture during the entire surgical procedure. Temperature was monitored using a rectal thermometer (Physitemp, TCAT‐2AC Controller) and controlled using a heating pad (Gaymar, T/Pump).

### 2.3 Functional activity assessment

Mouse functional activity was assessed at three timepoints: prestroke, 2 days poststroke (DPS), and 30 DPS. Mice were acclimatized to the behavior room for at least 30 min prior to testing. Then, animals were placed in individual environmental control chambers (Omnitech Electronics Inc, Columbus, OH) equipped with infrared beams crossing in the X, Y, and Z axes. Twenty‐four parameters of locomotor activity were assessed for each mouse over a 10‐min period. Data were collected using Fusion v5.3 software (Omnitech Electronics). The chamber was cleaned with Versaclean before and after each trial.

### 2.4 Magnetic resonance imaging and analysis

Magnetic resonance imaging (MRI) was used to assess brain infarct volume at two timepoints poststroke (2 and 30 DPS) using a Bruker Biospec 70/20 7.0T MRI scanner equipped with the ParaVision‐360.3.2 software (Bruker Biospin, Billerica, MA). An 86 mm ID volume coil was used for excitation and a four‐channel mouse brain phased array coil was used for reception. Mice were anesthetized with 1–3% isoflurane and 1 L/min of surgical oxygen and placed in a cradle equipped with a bite bar and ear bars. Throughout MRI, mouse body temperature was monitored via a fiber optic temperature probe and maintained at 37 ± 1°C using heated air, respiration rates were monitored using a pressure‐sensitive pad.

At 2 DPS, whole‐brain 3D T2‐weighted rapid acquisition with refocusing enhancement (RARE) images were acquired with TR/TE_effective_ = 600/64 ms, echo train length = 16, echo spacing = 8 ms, field of view = 25.6 mm (rostral–caudal) × 19.2 mm (left–right) × 12.8 mm (anterior–posterior), 200 μm isotropic resolution, 2 averages, scan time = 7:40 min:sec. Diffusion‐weighted MRI (DMRI) was also carried out in the coronal plane with 2D single‐shot echo‐planar imaging with reception time/echo time (TR/TE) = 5500/58 ms, field of view (FOV) = 18 mm × 18 mm, 250 × 250 μm (Thayabaranathan et al., 2022) in‐plane resolution, 0.8 mm slice thickness, 21 contiguous slices, 30 diffusion directions, b = 1600 s/mm (Thayabaranathan et al., 2022), δ = 5 ms, Δ = 40 ms, scan time = 3:02 min:sec. At 30 DPS, T2 and DMRI imaging were repeated and additional T1‐weighted RARE images were acquired before and after intraperitoneal injection of gadolinium‐based contrast agent (GBCA) MultiHance (Bracco Diagnostics Inc. Monroe Township, NJ) with TR/TE_effective_ = 600/8 ms, echo train length = 2, ESP = 8 ms, eight averages, FOV = 19.2 mm × 12.8 mm, 100 × 100 μm in‐plane resolution, 0.6 mm slice thickness, 21 contiguous slices, scan time = 5:07 min:sec. One T1‐weighted RARE image sequence was collected before intraperitoneal injection of GBCA, and three were collected thereafter at approximately 5‐min intervals.

Infarcts and hemispheric cross sections were manually delineated on T2‐weighted images using Mango version 4.1 (https://ric.uthscsa.edu/mango/index.html, Research Imaging Institute, UTHSCSA). Using hemispheric regions of interest, outlined in Mango software, edema and atrophy were calculated as a percent change in ipsilateral volume vs contralateral volume. Delineation was based on T2‐weighted signal hyperintensities. Apparent diffusion coefficient (ADC) and fractional anisotropy (FA) maps were created from diffusion‐weighted images using the MRTrix3 toolbox (Tournier et al., 2019) and the Tortoise pipeline (Pierpaoli et al., 2019). Briefly, DMRI images were reconstructed as 4D nifti volumes in BIDS‐compliant format using the BrkRaw Toolbox (BrkRaw v0.3.3) (Lee et al., 2020). Nifti images were preprocessed using the TORTOISE software package DIFFPREP, which corrects for motion, bias‐field, noise, Gibbs ringing, and eddy currents. Diffusion tensors were then computed from the preprocessed images using MRTrix3. ADC, FA, and diffusion‐encoded color (DEC) maps were calculated based on the tensors using standard procedures in MRTrix3. Percent‐enhancement maps were created for the 30 DPS timepoint using the T1‐weighted RARE sequences. Pre‐ and post‐GBCA injection T1‐weighted images were registered using an affine transformation in FMRIB Software Library (Jenkinson et al., 2012). The percent enhancement maps were calculated by dividing the registered GBCA‐enhanced T1‐weighted images by the registered pre‐GBCA‐enhanced T1‐weighted images on a voxel‐by‐voxel basis. The result was multiplied by 100 to get a percentage of GBCA enhancement from pre‐GBCA injection T1‐weighted images. The percent enhancement maps were generated using an in‐house Matlab program (Matlab R2023, The Mathworks Inc. Natick, MA). A T1 map was not acquired during the scan sequence, which precludes quantitative analysis of pharmacokinetic parameters related to BBB permeability.

### 2.5 Neurofilament light assessment

Immediately prior to sacrifice, plasma was collected to determine NFL concentrations for neuroaxonal injury assessment (Nielsen et al., 2020; Tiedt et al., 2018). Plasma samples were sent to PBL Assay Science (Piscataway, NJ, USA) and analyzed using the Simoa™ NF‐Light® kit (Cat no. 103186, Quanterix, Billerica, MA, USA) and platform.

### 2.6 Immunohistochemistry and tissue staining

Following MRI at 30 DPS, mice were sacrificed (overdose of isoflurane anesthetic agent, blood draw from cardiac puncture, followed by decapitation) and perfused with 0.01 M phosphate‐buffered saline. Brain tissue was removed and fixed in 4% paraformaldehyde for 24 hours followed by a 30% sucrose solution for 72 h and stored at −70°C prior to sectioning. Fixed tissue was sectioned and prepared as previously described (Young et al., 2021). A sampling of 5–7 coronal tissue sections (50 μm) per animal between bregma 1.5 and −4.0, to best span the infarct, was used for IHC and subsequent quantification of microglia morphologies as well as the area of phagocytic receptor CD68, CD220 (B‐cell), and CR45 (T‐cell) and BODIPY positive staining (Figure 1F). A free‐floating technique was used for IHC. For fluorescence IHC, brain sections were incubated for1 h in a blocking solution of 10% horse serum (Vector Laboratories, S‐2000‐20, Burlingame, CA) and buffer (0.01 M PBS, 0.05% Triton, and 0.04% NaN3) followed by a 72 h incubation with primary antibodies: rabbit anti‐IBA1 (1:1000, Wako, catalog no. 019‐19,741; RRID:AB_839504), rat‐anti CD68 (1:1000, Bio‐Rad Cat no. MCA1957GA, RRID:AB_324217), chicken‐anti GFAP (1:1000, Millipore Cat# AB5541, RRID:AB_177521), Syrian hamster‐anti CD3ε (1:1000; BD Biosciences, catalog no. 550277; RRID:AB_393573), B220/CD45R (1:500; BD Biosciences, catalog no. 553085; RRID:AB_394615). The tissue was then incubated for 4 hours with secondary antibodies: donkey anti‐rabbit Alexa 488 (Jackson ImmunoResearch Labs Cat# 711‐546‐152, RRID:AB_2340619) and donkey anti‐rat Alexa 594 (Jackson ImmunoResearch Labs Cat# 712‐585‐150, RRID:AB_2340688). All incubations took place at room temperature and in solutions common to all groups to avoid group/batch differences. Washes between incubations were with 0.01 M PBS for 15 min and Vectashield (Vector Laboratories, H‐1000) was used to coverslip mounted tissue. Nonfluorescence IHC was carried out on free‐floating sections using established standard protocols. Primary antibodies against CD3ε (1:1000; BD Biosciences, Cat. No. 550277; RRID:AB_393573) and B220/CD45R (1:500; BD Biosciences, Cat. No. 553085; RRID:AB_394615) were used with the appropriate secondary antibody and visualized using the VECTASTAIN Elite ABC Reagent, Peroxidase, R.T.U. (Vector Laboratories, Cat. No. PK‐7100) and Vector DAB Substrate (3,3′‐diaminobenzidine) Kit (Vector Laboratories, Cat. No. SK‐4100). Secondary antibodies were diluted 1:400 for biotinylated goat anti‐hamster IgG (Vector Laboratories, Cat. No. BA‐9100; RRID:AB_2336137), and biotinylated rabbit anti‐rat IgG (Vector Laboratories, Cat. No. BA‐4000; RRID:AB_2336206).

To visualize lipid droplets within cells and tissue of the infarct poststroke, free‐floating tissue sections were subjected to a boron‐dipyrromethene (BODIPY) 492/515 (ThermoFisher, D3922) staining protocol for fluorescence microscopy imaging and analysis using the manufacturer suggested protocol. Briefly, a working solution of BODIPY 492/515 was prepared by diluting 0.1 mg/mL BODIPY in dimethyl sulfoxide to a final concentration of 1:25. About 5–7 sections per animal, similar to those used for IHC, were rinsed in phosphate‐buffered saline and then incubated in the BODIPY solution for 30 min in a dark environment at room temperature followed by three 10‐min washes in PBS. Coverslips were applied using Vectashield HardSet Antifade mounting media (H‐1400) prior to image acquisition and analysis. IHC and BODIPY methods were combined to assess lipid droplet location within the infarct poststroke.

### 2.7 Optical image acquisition and analysis

For microglia morphology and CD68 percent area analysis, images were acquired on a confocal microscope (Zeiss NLO 880 equipped with Zen Black software, San Diego, CA) using a 40× objective (236.16 × 236.16‐micron area) in three cortex regions: contralateral, distal, and proximal to the injury. Microglia morphology was quantified from resulting 8‐bit images using previously described methods (Young & Morrison, 2018). Microglia morphology parameters (process length and number of endpoints) were summed, and all data were divided by the cell soma count per image frame to calculate the summed microglia process length/cell and endpoints/cell. Positive staining for CD68 was determined using consistent thresholding settings and ImageJ (vs.2.0.0‐rc‐69/1.52p, http://rsbweb.nih.gov/ij/). The percent area value of CD68‐positive staining in each image frame was divided by the microglia soma count for each image frame to achieve % area CD68/microglia cell. All data were averaged within each region for each animal for statistical analysis.

Fluorescent BODIPY staining and nonfluorescent images of B cells and T cells were acquired using a brightfield fluorescence microscope (10× or 20× objective; DMI6000) and stitched using LASX software. The area value of BODIPY‐, B220‐, or CD3e‐positive staining in the infarct was acquired using thresholding techniques to illuminate foam cells, B cells, or T cells. The percentage of BODIPY, B220, or CD3ε area in the ipsilateral hemisphere was acquired in multiple coronal sections for each brain (6–7 per mouse) and averaged to result in a single value, used for statistical analysis. Confocal microscopy was also used to describe the location of lipid droplets in higher magnification within the infarct using a 40× objective and 1.5 zoom. Unstained tissue was used to illustrate lipofuscin‐based autofluorescence that may be visualized in either the brightfield or confocal microscope acquisition.

### 2.8 Statistical analyses

The experimental unit for reported studies is a single mouse with sample sizes and statistical analysis reported within the results and/or figure legends. Data are presented as mean ± SD and *p* values less than 0.05 are considered statistically significant. As a descriptive study design, power analysis was not carried out; the sample size was based on previous experience concerning stroke survival in male and female mice with the goal of describing MRI and histological findings in male and female mice. Statistical analyses were performed using GraphPad Prism software 9.5.0 (GraphPad Software, La Jolla, CA, USA). Normality was assessed using the Shapiro–Wilk tests, and *t*‐tests and ANOVA were used to compare groups. Principal component analysis (PCA) analysis was carried out on functional activity using R and RStudio. No available data were excluded from analysis.

## 3 RESULTS

**FIGURE 2:**
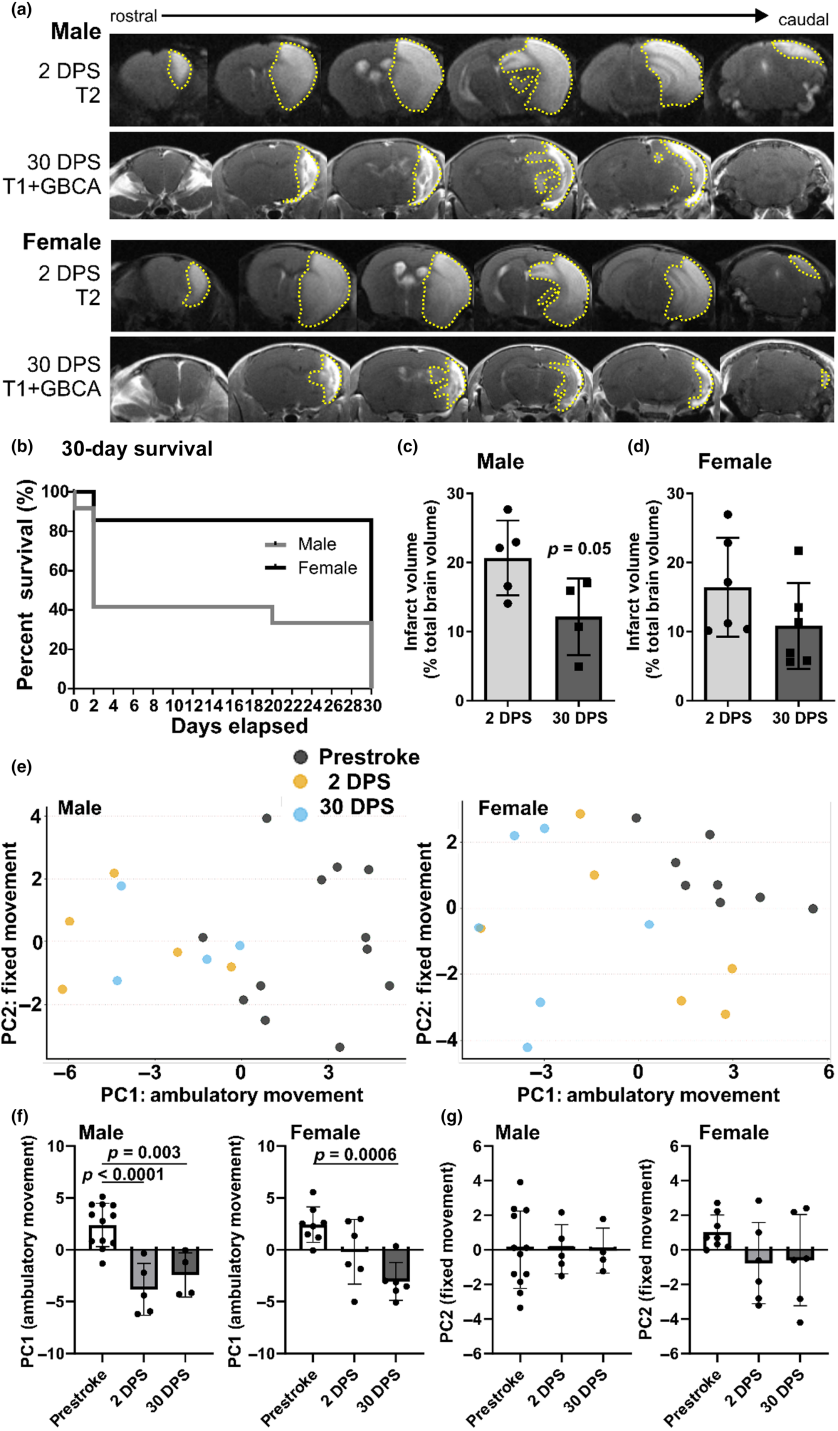
Poststroke survival, infarct volume, and functional activity at 2 and 30 days poststroke (DPS). Male and female mice underwent 45 min of focal ischemic stroke followed by recovery. (a) Brain tissue in male and female mice at 2 DPS imaged using T2 RARE with increased signal intensity (gray) that is indicative of poststroke infarct volume (outlined in yellow dashed line) in male and female mice. Brain tissue in male and female mice at 30 DPS imaged using T1 imaging after gadolinium (GBCA) injection, helpful to visualize poststroke infarct volume (outlined in yellow dashed line) at 30 DPS. (b) Summary data of 30‐day survival curves in male and female mice with male mice being more impacted by attrition than female mice (*Χ* ^2^_1,19_ = 4.46, *p* = 0.03; male *n* = 12, female *n* = 7). Summary data of poststroke infarct volume at 2 DPS, and 30 DPS in male (c) and female mice (d) illustrate a 40% (*p* = 0.05) and 34% decrease in infarct volume by 30 DPS, respectively. (e) Scatter plots of PC1 and PC2 summarizing functional activity assessed prestroke, 2 DPS and 30 DPS in male and female mice. PC1 summarized locomotor movements crossing X, Y, and Z planes (labeled ambulatory movement), and PC2 summarized stereotype movement behaviors (labeled fixed movement). (f) Summary data of PC1 illustrating significant changes in functional activity as early as 2 DPS in male but not female mice; functional activity was changed versus prestroke assessment at 30 days in both male and female mice. All *p*‐values are reported within the figure. (g) The PC2 variable includes data such as summary data of PC2 illustrating no significant changes in activity in male or female mice at 2 DPS or 30 DPS.

Male (*n* = 12) and female (*n* = 7) mice underwent 45 min of focal ischemic stroke followed by 30‐day recovery. Representative MRI images used for infarct volume analysis are shown in Figure 2a. Survival assessment showed that 86% of female mice and 42% of male mice survived to 2 DPS for MRI assessment of infarct volume and 33% of male mice survived to 30 DPS, whereas there were no additional female deaths occurring past 2 DPS (Figure 2b). Infarct volume was assessed in all surviving male (Figure 2c) and female (Figure 2d) mice at 2 DPS and 30 DPS to illustrate infarct volume resorption over time (male *n* = 4: 40% decrease, *p* = 0.05; female *n* = 6: 34% decrease, *p* > 0.05).

Functional activity was assessed prestroke, 2 DPS, and 30 DPS in surviving mice using environmental control chambers to measure 24 parameters of motor activity. PCA was carried out for data from male and female groups to reduce the multiple locomotor parameters into two new measurements of functional activity, PC1 and PC2. In male and female mice, primary locomotor variables that contributed to PC1 were ambulatory activity count and movement, and as such, PC1 was labeled as “ambulatory movement.” For males, the main variables contributing to PC2 were stereotypic movements that resemble head bobbing and grooming behaviors, distinct from typical locomotor movement, and therefore, PC2 was identified as stereotypic or “fixed movement.” Only slightly different in females, primary variables for PC2 included rest episodes and moment episode counts. In summary, while PC1 summarized locomotor movements crossing X, Y, and Z planes, PC2 summarized fixed movement behaviors. The relationship between PC1 and PC2 in male and female mouse groups are shown (Figure 2e). In males, there was an immediate (2 DPS) and lasting (30 DPS) decrease in locomotor activities, summarized by PC1 (*p* < 0.0001 and *p* < 0.01). In females, locomotor activity was decreased at 30 DPS (*p* < 0.001; Figure 2f). On the other hand, stereotypic activities, summarized by PC2, were unchanged poststroke in male and female mice (Figure 2g).

**FIGURE 3:**
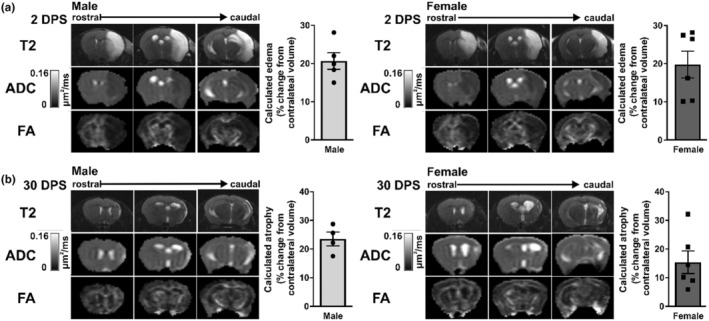
Magnetic resonance imaging findings at 2 and 30 days poststroke (DPS). Representative axial views of T2, apparent diffusion coefficient (ADC), and fractional anisotrophy (FA) maps acquired at 2 DPS (a) and 30 DPS (b) in male and female mice with accompanying summary data of edema and atrophy calculated from T2 images. Hyperintensities in T2 images represent increased cellular water uptake while hypointensities in ADC and FA images represent restricted water diffusion in all diffusion directions. ADC scale bar = μm^2^/ms, with low microscopic water diffusion (black) to high microscopic water diffusion (white).

Acute and chronic brain injury at 2 DPS and 30 DPS, respectively, were assessed via infarct volume and water diffusion using MRI. At 2 DPS, cytotoxic and vasogenic edema was clearly seen in both male and female mice (Figure 3a). The cellular swelling associated with ischemic stroke was observed as a hyperintensity in the T2‐weighted image where the associated tissue swelling caused an observable midline and ventricle shift in both male and female mice. Edema resulting from injury was calculated as a percent increase in ipsilateral volume from contralateral volume using the 2DPS T2 images (Figure 3a). Diffusion MRI (DMRI) was also collected at 2 DPS, with the ADC and FA results shown in Figure 3a. In the injury region, ADC was decreased due to cytotoxic and vasogenic edema in male and female mice. Brain microstructure remained unaffected contralateral to the region of stroke in both male and female mice. The vasogenic and cytotoxic edema in the region of the stroke also greatly impacted FA (Figure 3a). White matter and regions of high anisotropy remained unchanged in the contralateral region, but anisotropy could not be accurately determined ipsilaterally due to increased cellular swelling.

T2, ADC, and FA at 30 DPS are shown in Figure 3b. Structural changes and remodeling after stroke were observed in the T2‐weighted images. Increased ventricle volume ipsilateral and contralateral indicated a loss of brain tissue volume and an increase in cerebrospinal fluid (CSF) volume. The T2‐weighted images also showed a region of hyperintensity ipsilateral to the infarct, indicating free‐diffusing fluid had replaced tissue in both the male and female mice. Additionally, a midline shift was observed in both the male and female mice in the T2‐weighted images, but now due to the loss of tissue, rather than the tissue swelling observed at 2 DPS. Atrophy was calculated as a percent decrease in ipsilateral volume from contralateral using 30 DPI T2 images (Figure 3b). The ADC in Figure 3b indicated a shift from cytotoxic edema to vasogenic edema compared to the 2 DPS time point as well as a region of hyperintensity and increased apparent diffusion ipsilateral to the ischemia. FA in male and female mice at 30 days in Figure 3b indicated that the majority of the microstructure remained intact in the contralateral and ipsilateral regions. Increased anisotropy along the edge of the ischemic region indicated a change in structure compared to the contralateral side.

**FIGURE 4:**
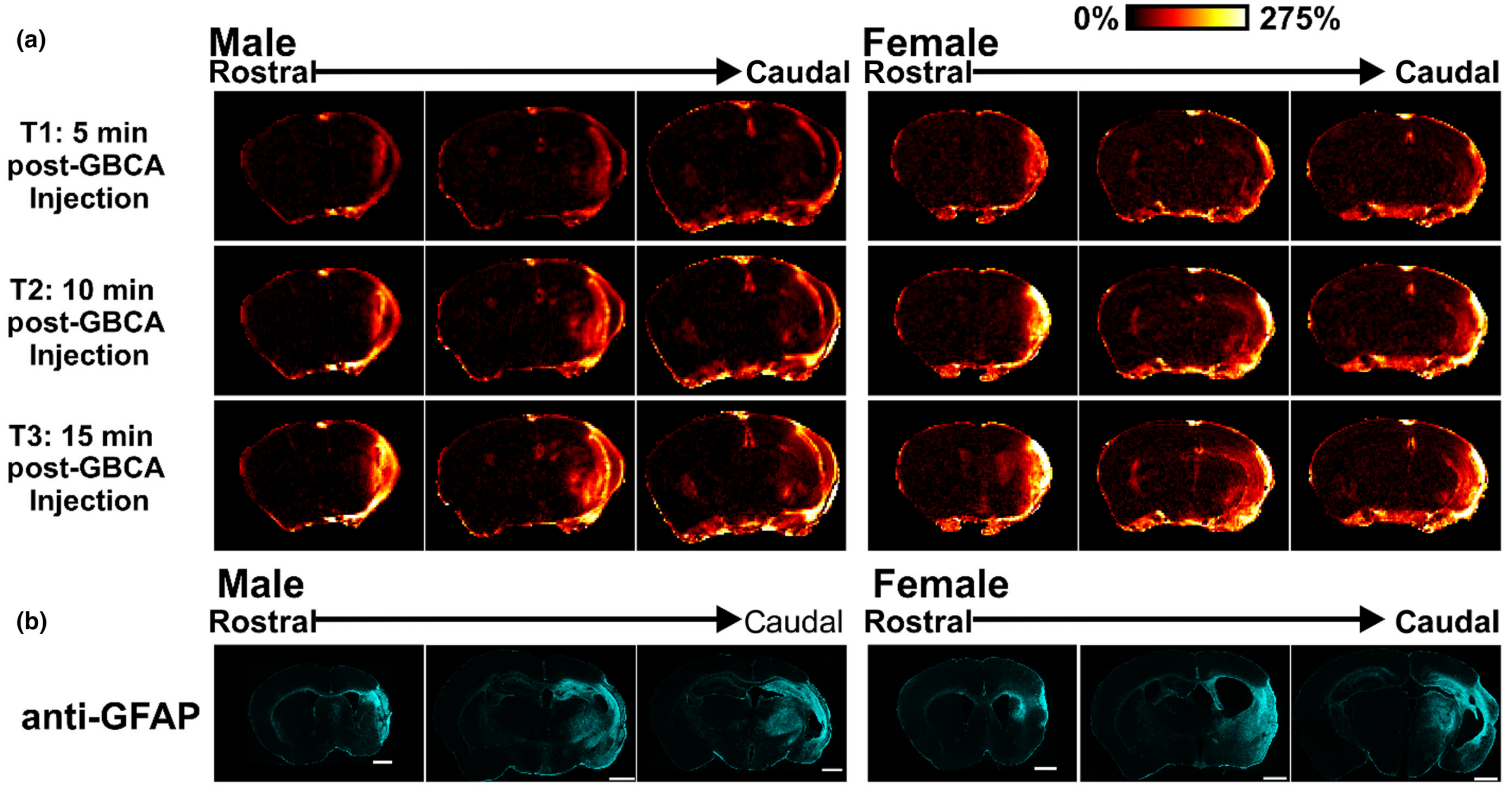
Gadolinium leakage corresponds with chronic gliosis describing poststroke chronic brain injury. (a) Gadolinium (GBCA) detection using T1 magnetic resonance imaging identifies the area of tissue resorption and resulting “dead space” within the ipsilateral hemisphere as evidenced by intense hyperintensity (bright yellow/white). Red, orange, and yellow colors illustrate gadolinium diffusion as a % enhancement from the pre‐GBCA T1‐RARE sequence (0%–275%) within the infarct and ipsilateral hemisphere with increasing time (T1–T3; 15 min). (b) Representative images of anti‐GFAP fluorescence immunohistochemistry were used to identify gliosis and the presence and location of a poststroke astrocyte glial scar.

The leakage of gadolinium across the BBB over time was used as an additional descriptor of chronic brain injury at 30 DPS. Figure 4a shows the percent enhancement of signal in T1‐weighted images at 3 times postinjection (5‐min intervals). The bright signal (white/yellow) abutting the skull in the ipsilateral hemisphere reflects the gadolinium quickly diffusing into the infarct but also highlights the gap between tissue and skull that results from tissue resorption and cavitation. Lesser quantities of gadolinium, observed as red/orange, diffused into the ipsilateral hemisphere with the amount increasing over time—an observation that illustrates the permeability of the glial scar that surrounds the infarct in the ipsilateral hemisphere. GFAP visualization of the astrogliosis in the ipsilateral hemisphere mirrored the MRI findings of the chronic brain injury at 30 DPS (T2, ADC, FA, and T1‐Gd) (Figure 4b).

**FIGURE 5:**
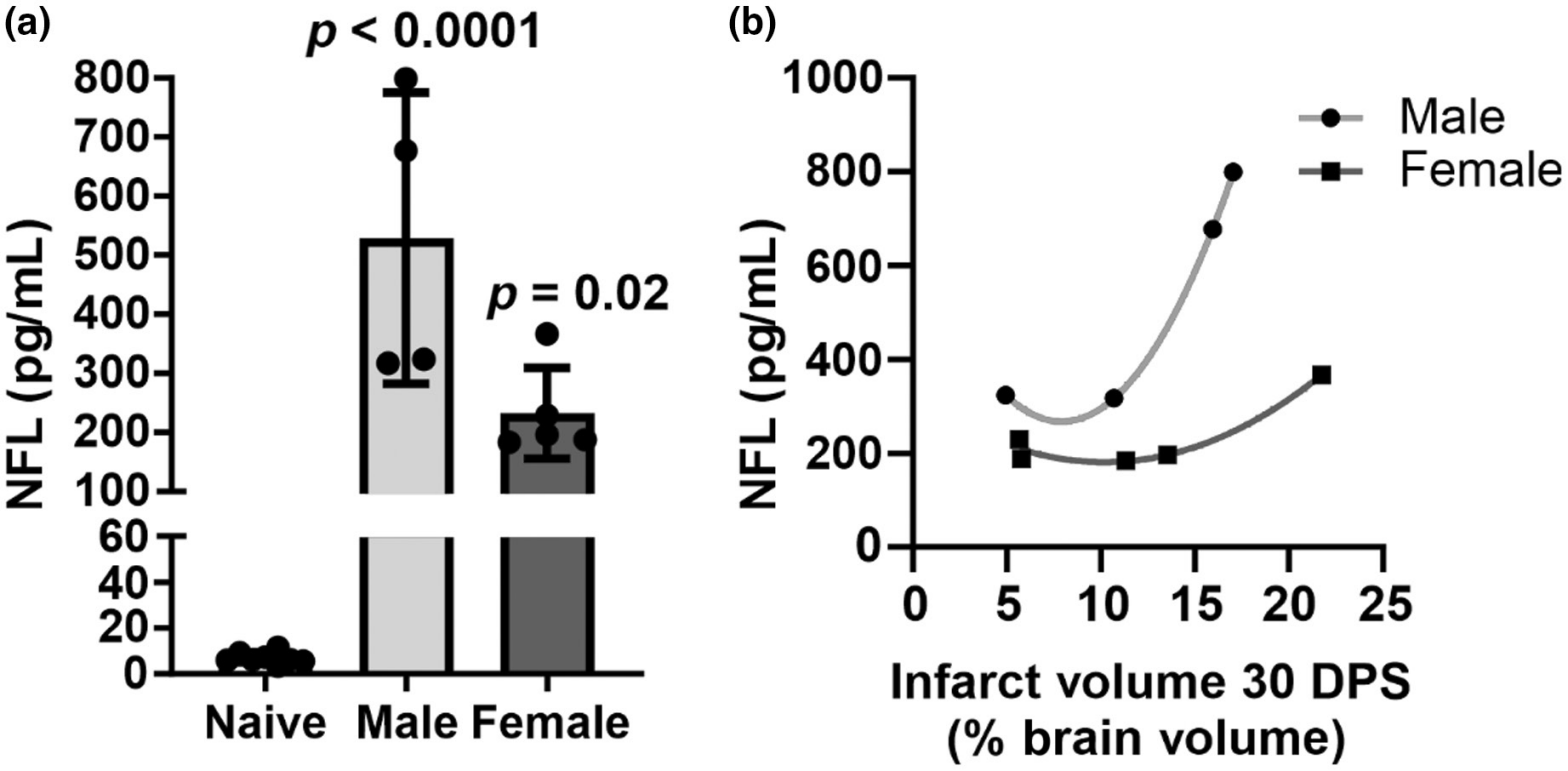
Neurofilament light (NFL) concentrations are increased in male and female mice at 30 days poststroke (DPS). (a) Summary data of NFL concentrations in male (*n* = 4) and female (*n* = 5) mouse plasma at 30 DPS (ANOVA, *p* values versus naïve reported in the figure). (b) Summary data of 30 DPS NFL data and infarct volume correlation illustrate a significant nonlinear relationship when stratified by sex (nonlinear regression, male: *r* = 0.99; female: *r* = 0.98).

Neurofilament light concentrations were measured as a biomarker of neuroaxonal injury in male and female mice with ischemic stroke. Naïve mice were used as a control instead of sham which may limit the interpretation of findings. NFL concentrations remained elevated at 30 DPS (vs. naïve, male, *p* < 0.0001; female, *p* < 0.05; Figure 5a). A significant and nonlinear relationship was observed between infarct volume and NFL concentrations (Figure 5b, male: *r* = 0.99; female: *r* = 0.98).

**FIGURE 6:**
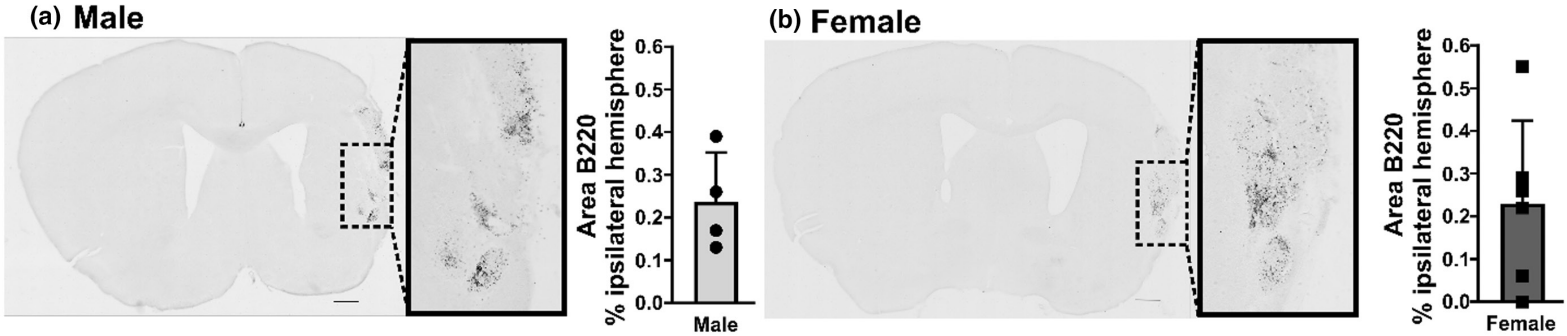
B lymphocytes populate the infarct in male and female mice at 30 days poststroke (DPS). (a) Stitched images of B220 (B lymphocytes) immunostaining in tissue from male (a) and female (b) mice along with summary data of the measured percent area of B220 immunoreactivity within the ipsilateral hemisphere at 30 DPI (male *n* = 4; female *n* = 6). Scale bar: 500 μm.

**FIGURE 7:**
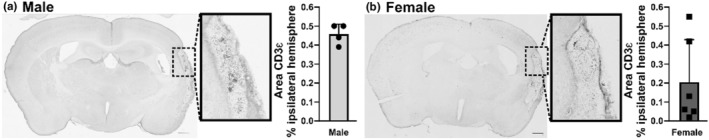
T cells populate the infarct in male and female mice at 30 days poststroke (DPS). Stitched images of CD3ε (T cells) immunostaining in tissue from male (a) and female (b) mice at 30 DPI and summary data of the measured percent area of CD3ε immunoreactivity within the ipsilateral hemisphere (male *n* = 4; female *n* = 6). Scale bar: 500 μm.

Figure 6 illustrates that B cells are present in the chronic infarct at 30 DPS in male and female mice. Similarly, T cells are also present in the chronic infarct at 30 DPS in male and female mice (Figure 7). There was a strong association between the area of B220 immunoreactivity in the ipsilateral hemisphere and infarct size at 30 DPI (*r* = 0.46), but a small association between the area of CD3e immunoreactivity in the ipsilateral hemisphere and infarct size (*r* = 0.19).

**FIGURE 8:**
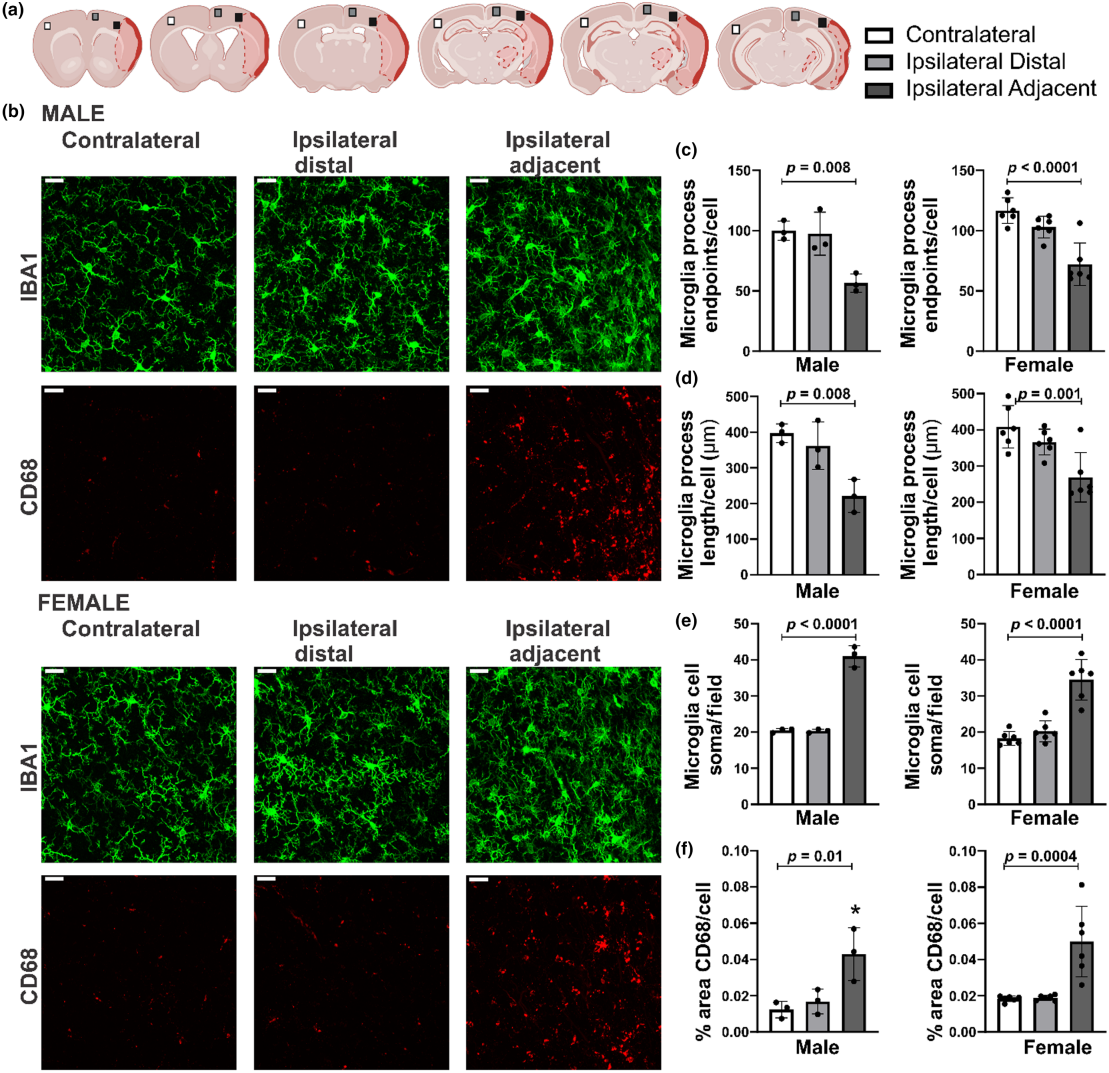
Microglia responses at 30 days poststroke (DPS) are present only adjacent to the infarct. (a) An illustration of the regions imaged in progressive brain slices sampled from rostral to caudal. (b) Images of IBA1‐positive microglia (40× objective) and CD68 in three cortical regions in proximity to the infarct (contralateral, ipsilateral distal, and ipsilateral adjacent) in male and female mice at 30 DPS. Summary data of skeleton analysis show that both the number of microglia process length/cell (c) and summed process endpoints/cell (d) decreases in proximity to the brain injury in male and female mice (*p* values reported in the figure). (e) Summary data of microglia soma counts show that the number of microglia in images collected from the ipsilateral adjacent region is increased versus the contralateral region in male and female mice (*p* values reported in the figure). (f) Summary data of the percent area of CD68 immunofluorescence per microglia cell is increased adjacent to the brain injury versus contralateral region (*p* values reported in the figure). Male *n* = 3; female *n* = 6 for all regions. Scale bar: 20 μm.

Microglia responses in the ipsilateral cortex at 30 DPS in surviving male and female mice were assessed. Figure 8a shows the brain regions imaged using a 40× objective in relation to the infarct. Figure 8a,b illustrates the Iba1‐positive microglia and CD68 immunofluorescence imaged in each brain region in male and female mice. The summary of microglia morphology data illustrates that microglia were de‐ramified in the region adjacent to the stroke injury at 30 DPS. Microglia process endpoints/cell were markedly reduced adjacent to the injury but not in the more distal region in male and female mice (Figure 8c, male: *F* = 12.21, *p* = 0.008; female: *F* = 18.59, *p* < 0.0001; post hoc comparisons vs. contralateral region are reported in figure) as were microglia process length/cell (Figure 8d, male: *F* = 10.87, *p* = 0.01; female: *F* = 9.81, *p* = 0.002; post hoc comparisons vs. contralateral are reported in figure). Approximate number of microglia residing in the adjacent region was nearly double that of the distal or contralateral regions in male and female mice (Figure 8e; male: *F* = 137.4, *p* < 0.0001; female: *F* = 32.3, *p* < 0.0001; post hoc comparisons vs. contralateral are reported in the figure). Last, the percent area of positive CD68 immunofluorescence per cell was increased in the region adjacent to the injury but not the more distal region in male and female mice (Figure 8f; male: *F* = 8.78, *p* = 0.02; female: *F* = 15.27, *p* < 0.0002; post hoc comparisons vs. contralateral are reported in the figure). Taken together, these data indicate that microglia responses, indicated by decreased microglia ramification and increased CD68 expression, are localized to the tissue region adjacent to the infarct when assessed at 30 DPS.

**FIGURE 9:**
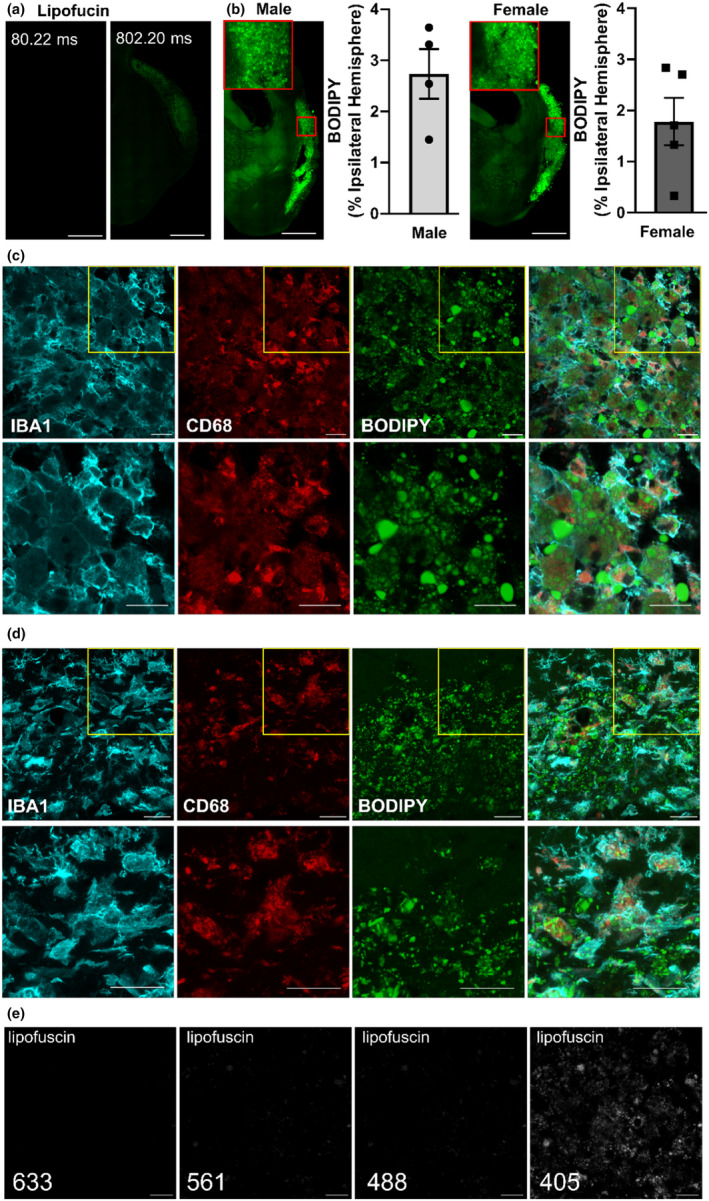
Lipid‐rich foam cells are abundant in the infarct at 30 days poststroke (DPS) in male and female mice. (a) Unstained tissue was imaged to illustrate underlying lipofuscin‐based autofluorescence. Images were obtained using exact power and exposure settings (80.22 ms) as images shown in boron‐dipyrromethene (BODIPY) staining (B) and again at 802.20 ms exposure. Scale bar: 1000 μm. (b) Representative images of BODIPY in the infarct at 30 DPS with summary data of BODIPY area in male (*n* = 4) and female (*n* = 5) mice. Scale bar: 1000 μm. (c) A representative image in a lipid‐rich region of the infarct using immunohistochemistry and BODIPY to illustrate a clustering of foam cells (IBA1/CD68) with intracellular and extracellular lipids. Scale bar: 20 μm (d) A representative image of a mixed population of foam cells (IBA1/CD68 cells with intracellular) and microglia/macrophages (IBA1/CD68 cells without BODIPY) in infarct. Scale bar: 20 μm. (e) Unstained tissue imaged to illustrate underlying lipofuscin‐based autofluorescence with lasers used for confocal imaging acquisition shown in “c” and “d.” Lipofuscin‐based autofluorescence is visible using the 405 laser (commonly used for DAPI acquisition) but not when using the 488, 561, or 633 lasers (acquisition settings the same as “d” and “e”). These images illustrate that artifacts from cell debris in the chronic infarct do not interfere with images acquired for “d” and “e.”

The presence of foam cells in the infarct of male and female mice at 30 DPS was investigated using BODIPY staining as shown in Figure 9. In Figure 9a, we show that an exposure time of 802.20 ms is necessary to visualize lipofuscin autofluorescence in unstained stroke tissue. In contrast, an exposure time of 80.22 ms revealed no lipofuscin autofluorescence in unstained tissue but this shorter exposure time was sufficient to detect BODIPY fluorescence in the infarct. In Figure 9b, representative images of BODIPY fluorescence within the infarct at 30 DPS are shown, along with summary data quantifying BODIPY fluorescence in both male and female mice. Additionally, we employed a combination of IHC and BODIPY staining to localize the presence of BODIPY fluorescence to microglia/macrophages within two regions of the post‐stroke infarct. Figure 9c depicts an area consisting solely of foam cells, whereas Figure 9d shows a tissue region with a combination of microglia/macrophages and foam cells. In Figure 9e, we present images of unstained tissue captured under the same confocal settings as used for Figure 9c,d. This was done to verify the absence of autofluorescence originating from lipofuscin in Figure 9d,e. Furthermore, lipofuscin based autofluorescence is visible using the 405 laser (commonly used for DAPI acquisition) but not when using the 488, 561, or 633 lasers (acquisition settings the same as D and E). These images illustrate that artifacts from cell debris in the chronic infarct do not interfere with images acquired for D and E.

## 4 DISCUSSION

The challenges facing the progression of stroke therapies into clinical trials are multifaceted (Lyden et al., 2022, 2023). Among these challenges is the insufficient understanding of the intricacies and trajectory of stroke recovery, with a predominant emphasis on acute rather than chronic timepoints. While an acute study design is well suited for evaluating drug efficacy in mitigating the initial brain injury resulting from ischemia, it tends to overlook an alternative therapeutic approach—interventions aimed at enhancing recovery. With this perspective in mind and as a central focus of our work, we aimed to document the long‐term consequences of ischemic stroke using MRI and histology techniques in male and female mice in the most used reperfusion model of ischemic stroke. Considering this focus, the subacute phase of poststroke injury evolution was not included as a study objective and limits the scope of our findings.

Notable findings from this study include the sensitivity of NFL as an indicator of post‐stroke neuronal injury. MRI assessments at 2 DPS revealed an increase in both vascular edema and cytotoxic edema. MRI at 30 DPS demonstrated significant brain tissue atrophy on the ipsilateral side resulting from the stroke, with the midline shifting toward the ipsilateral side. It also revealed that the BBB is still compromised at 30 DPS. Concerning neuroinflammation, we observed the presence of both B cells and T cells within the infarct, noting that microglia responses during the chronic poststroke phase are primarily localized to the region immediately adjacent to the infarct rather than spanning the entirety of the ipsilateral hemisphere. Last, we reported on the transformation of microglia/macrophages into lipid‐laden foam cells within the infarct at 30 DPS.

A limitation of this study is the survival rate of male mice, which was particularly low at the 2‐day MRI assessment, although they survived the initial 24–36 h. This pattern, consistent with findings from other studies (Dirnagl, 2016; Macrae, 2011), is attributed to infarct sizes exceeding 25% of total brain volume, as confirmed by necropsy. To facilitate longitudinal studies, it may be necessary to adjust the duration of ischemia time, (e.g., from 30 to 45 min) to ensure a more survivable poststroke condition in male mice. On the other hand, shortening the duration of ischemia may introduce variability in poststroke MRI outcomes (infarct volume) and limit the detection of functional behavior changes due to the milder injury. The early attrition of male mice, resulting in a smaller sample size for this group, reflects the broader sex differences in stroke survival noted in other research (Ahnstedt et al., 2016).

Our assessment of poststroke functional behavior primarily employed an automated locomotor assessment, which measures multiple variables related to mouse movement. This approach minimizes animal handling and reduces bias (Mingrone & Kaffman, 2020). Cognitive function was not assessed which is a limitation of this study. Principal component analysis (PCA) was used to reduce the dimensionality of the functional behavior data into two principal components (PC1 and PC2) that effectively summarized the complex functional activity behavior into two behavior types, ambulatory movement and stereotypic or fixed movement. This approach allowed us to leverage the entire data set to detect deficits in mouse locomotor activity poststroke, increasing sensitivity by capturing all variability in the data while reducing bias and redundancy in reporting.

Neurofilament light is a neuronal cytoskeletal protein released into the CSF and found in blood, enabling objective and precise assessment of neuronal injury and recovery over time (Nielsen et al., 2020; Tiedt et al., 2018). Accurately assessing smaller infarcts via MRI at chronic timepoints can be challenging, as it may not reliably visualize the infarct. This underscores the importance of having an objective measure for neuronal injury, with NFL emerging as a promising biomarker. Clinical findings also support the significance of NFL, as it has been reported to correlate with stroke severity and functional outcomes in stroke patients (Nielsen et al., 2020).

Magnetic resonance imaging assessments at 2 DPS revealed an increase in both vasogenic edema (evidenced by increased hyperintensity in T2‐weighted images) and cytotoxic edema (indicated by decreased ADC), which is typical of large brain injuries at acute poststroke timepoints (Osa García et al., 2022). This edema leads to a mass effect, causing the midline to bulge toward the contralateral side of the brain, a phenomenon well documented in experimental and clinical settings (Dhar et al., 2016; McKeown et al., 2022; Yoo et al., 2013). Additionally, a loss of diffusion anisotropy along white matter tracts on the ipsilateral side is observed due to this edema. MRI at 30 DPS demonstrated significant brain tissue atrophy on the ipsilateral side resulting from the stroke, with the midline shifting toward the ipsilateral side as brain tissue is replaced by CSF. The enlarged ventricles on the ipsilateral side, observed in T2‐weighted images and ADC maps, reflect the loss of brain tissue and its replacement with CSF. These findings underscore the importance of assessing ventricle size, as recently endorsed by SPAN, as a poststroke injury variable (Lyden et al., 2022). Fractional anisotropy maps indicate a pseudonormalization of the remaining white matter in regions due to a loss of membrane structure and vasogenic edema (Mandeville et al., 2017). This suggests that microscopic tissue changes, such as cytotoxic versus vasogenic edema, cannot be accurately resolved based solely on DMRI (Osa García et al., 2022). These MRI data are critical because, without contrast‐enhanced imaging, one might infer that the remaining brain tissue on the ipsilateral side is normal. However, contrast‐enhanced MRI shows that this is not the case, as the BBB on the ipsilateral side is highly permeable to GBCA. These findings validate previous data collected using alternative methods (Zbesko et al., 2018).

The presence and distribution of B cells and T cells within the infarct poststroke are well documented in various stroke models and timepoints (Ahnstedt et al., 2020; Doyle et al., 2015; Ito et al., 2019; Vindegaard et al., 2017; Zbesko et al., 2020). In our study, we confirmed these findings in the MCAO model with reperfusion, including both male and female mice, with no sex effect observed. An examination of the relationship between infarct size and the accumulation of B cells suggested a significant connection, although this may not hold true for T cells. Although this study was not specifically powered to detect small sex effects, others have noted a sex‐specific increase in infiltrating CD8+ T cells in males but not CD4+ T cells, accompanied by increased circulating blood CD8+ T cells and CD8/CD4 ratios (Ahnstedt et al., 2020). The literature indicates that the effects of T cells in ischemic injury may vary depending on their classification (e.g., Treg, Th1, Th2, etc.) (Ito et al., 2019; Liesz et al., 2009). Therefore, additional efforts to classify T‐cell subtypes in male and female models at chronic poststroke timepoints are needed to identify potential therapeutic targets for reducing poststroke complications, including those influenced by sex, such as dementia.

In previous studies, we have documented widespread morphologic and functional responses of microglia in the ipsilateral hemisphere at acute timepoints (Morrison & Filosa, 2013, 2016; Young et al., 2021). At 30 DPS, numerous microglia with de‐ramified morphologies and the presence of CD68, a lysosomal scavenger protein, were primarily confined to the region immediately adjacent to the infarct, within approximately 250 μm of the injury site. In contrast, at a more distal region (>500 μm distance), microglia morphology and CD68 expression resembled those in the contralateral hemisphere. Additionally, at a 24‐h poststroke timepoint, microglia near the stroke injury exhibited highly phagocytic behavior characterized by the presence of ball‐shaped morphologies and phagosomes, which were not observed at 30 DPS (Young et al., 2021). Microglia are exceptionally responsive to their environment as indicated by diverse morphological changes (Sierra et al., 2013; Tremblay et al., 2011), and morphology can provide early indications of microglial response to injury (Morrison et al., 2017; Morrison & Filosa, 2016). Therefore, these data could suggest that, compared to acute timepoints, there is some resolution or containment of poststroke inflammation in the ipsilateral hemisphere at 30 DPS.

Poststroke wound healing in the brain occurs in a lipid‐rich environment, with microglia and macrophages acting as resident and systemic phagocytes responsible for engulfing and processing lipids resulting from myelin breakdown as neurons undergo poststroke necrosis. Consistent with findings from stroke models without reperfusion (Chung et al., 2018), our research in the transient MCAO model with reperfusion also demonstrates that microglia and macrophages transform into foam cells. While foam cells are commonly associated with atherosclerosis, they are also present in the brain of individuals with Niemann–Pick disease, a heritable disorder characterized by an inability to metabolize lipids (Schuchman & Desnick, 2017). Strategies to treat Niemann–Pick may hold promise as a relevant treatment post‐stroke to facilitate lipid clearance and improve brain wound healing processes.

## 5 CONCLUSIONS

In conclusion, our study provides valuable insights into the chronic phase of ischemic stroke recovery, documenting various long‐term consequences in the commonly used mouse model of transient MCAO. By comprehensively assessing stroke outcomes in male and female mice using advanced imaging and histological techniques, we have contributed to the growing body of knowledge in stroke research. These findings emphasize the potentially critical roles that persistent inflammation, stemming from the lipid‐rich brain environment primarily due to myelin, and the ongoing disruption of the BBB. These factors may significantly contribute to the development of secondary neurodegeneration. We show that poststroke injury is chronic and multifaceted highlighting the need for further investigations to advance stroke therapies and improve outcomes for stroke survivors.

## AUTHOR CONTRIBUTIONS

Conceptualization: HWM and TPT; Investigation: all authors; Methodology: HWM and TPT; Software: DPM and TPT; writing (original draft preparation): HWM and KPD; writing (review and editing): DPM, DSD, HWM, KPD, and TPT; supervision: HWM and TPT; project administration: HWM; funding acquisition: HWM and TPT.

## FUNDING INFORMATION

This research was funded by NINDS 1R41NS124450 (HWM and TPT), NIA T32AG082631‐01 (DPM), S10 OD025016 (Bruker 7T).

## CONFLICT OF INTEREST STATEMENT

The authors declare that they have no competing or conflicts of interests.

## ETHICS APPROVAL AND CONSENT TO PARTICIPATE

The animal study protocol was approved by the Institutional Animal Care and Use Committee (IACUC) of the University of Arizona.

## Data Availability

The data sets used and/or analyzed during the current study are available from the corresponding author on reasonable request.

## References

[phy216118-bib-0001] Adigun, R. , Basit, H. , & Murray, J. (2023). StatPearls. StatPearls Publishing. 28613685

[phy216118-bib-0002] Ahnstedt, H. , McCullough, L. D. , & Cipolla, M. J. (2016). The importance of considering sex differences in translational stroke research. Translational Stroke Research, 7, 261–273. 10.1007/s12975-016-0450-1 26830778 PMC4929018

[phy216118-bib-0003] Ahnstedt, H. , Patrizz, A. , Chauhan, A. , Roy‐O'Reilly, M. , Furr, J. W. , Spychala, M. S. , D'Aigle, J. , Blixt, F. W. , Zhu, L. , Bravo Alegria, J. , & McCullough, L. D. (2020). Sex differences in T cell immune responses, gut permeability and outcome after ischemic stroke in aged mice. Brain, Behavior, and Immunity, 87, 556–567. 10.1016/j.bbi.2020.02.001 32058038 PMC7590503

[phy216118-bib-0004] Banerjee, A. , & McCullough, L. D. (2022). Sex‐specific immune responses in stroke. Stroke, 53, 1449–1459. 10.1161/strokeaha.122.036945 35468002 PMC9668253

[phy216118-bib-0005] Becktel, D. A. , Zbesko, J. C. , Frye, J. B. , Chung, A. G. , Hayes, M. , Calderon, K. , Grover, J. W. , Li, A. , Garcia, F. G. , Tavera‐Garcia, M. A. , Schnellmann, R. G. , Wu, H. J. J. , Nguyen, T. V. V. , & Doyle, K. P. (2022). Repeated administration of 2‐hydroxypropyl‐β‐cyclodextrin (HPβCD) attenuates the chronic inflammatory response to experimental stroke. The Journal of Neuroscience, 42, 325–348. 10.1523/jneurosci.0933-21.2021 34819339 PMC8802936

[phy216118-bib-0006] Chung, A. G. , Frye, J. B. , Zbesko, J. C. , Constantopoulos, E. , Hayes, M. , Figueroa, A. G. , Becktel, D. A. , Antony Day, W. , Konhilas, J. P. , McKay, B. S. , Nguyen, T. V. V. , & Doyle, K. P. (2018). Liquefaction of the brain following stroke shares a similar molecular and morphological profile with atherosclerosis and mediates secondary neurodegeneration in an Osteopontin‐dependent mechanism. eNeuro, 5, ENEURO.0076‐18.2018. 10.1523/eneuro.0076-18.2018 PMC6223114 30417081

[phy216118-bib-0007] Dhar, R. , Yuan, K. , Kulik, T. , Chen, Y. , Heitsch, L. , An, H. , Ford, A. , & Lee, J. M. (2016). CSF volumetric analysis for quantification of cerebral edema after hemispheric infarction. Neurocritical Care, 24, 420–427. 10.1007/s12028-015-0204-z 26438467 PMC4821820

[phy216118-bib-0008] Dirnagl, U. (2016). Rodent models of stroke. Springer.

[phy216118-bib-0009] Doyle, K. P. , & Buckwalter, M. S. (2017). Does B lymphocyte‐mediated autoimmunity contribute to post‐stroke dementia? Brain, Behavior, and Immunity, 64, 1–8. 10.1016/j.bbi.2016.08.009 27531189 PMC5305803

[phy216118-bib-0010] Doyle, K. P. , & Buckwalter, M. S. (2020). Immunological mechanisms in poststroke dementia. Current Opinion in Neurology, 33, 30–36. 10.1097/WCO.0000000000000783 31789707 PMC7251986

[phy216118-bib-0011] Doyle, K. P. , Quach, L. N. , Solé, M. , Axtell, R. C. , Nguyen, T. V. V. , Soler‐Llavina, G. J. , Jurado, S. , Han, J. , Steinman, L. , Longo, F. M. , Schneider, J. A. , Malenka, R. C. , & Buckwalter, M. S. (2015). B‐lymphocyte‐mediated delayed cognitive impairment following stroke. The Journal of Neuroscience, 35, 2133–2145. 10.1523/JNEUROSCI.4098-14.2015 25653369 PMC4315838

[phy216118-bib-0012] Droś, J. , & Klimkowicz‐Mrowiec, A. (2021). Current view on post‐stroke dementia. Psychogeriatrics, 21, 407–417. 10.1111/psyg.12666 33608997

[phy216118-bib-0013] Ito, M. , Komai, K. , Mise‐Omata, S. , Iizuka‐Koga, M. , Noguchi, Y. , Kondo, T. , Sakai, R. , Matsuo, K. , Nakayama, T. , Yoshie, O. , Nakatsukasa, H. , Chikuma, S. , Shichita, T. , & Yoshimura, A. (2019). Brain regulatory T cells suppress astrogliosis and potentiate neurological recovery. Nature, 565, 246–250. 10.1038/s41586-018-0824-5 30602786

[phy216118-bib-0014] Jenkinson, M. , Beckmann, C. F. , Behrens, T. E. , Woolrich, M. W. , & Smith, S. M. (2012). FSL. NeuroImage, 62, 782–790. 10.1016/j.neuroimage.2011.09.015 21979382

[phy216118-bib-0015] Lee, S.‐H. , Ban, W. , & Shih, Y.‐Y. I. (2020). BrkRaw/bruker: BrkRaw v0.3.3 (0.3.3) . Zenodo. 10.5281/zenodo.3877179

[phy216118-bib-0016] Liesz, A. , Suri‐Payer, E. , Veltkamp, C. , Doerr, H. , Sommer, C. , Rivest, S. , Giese, T. , & Veltkamp, R. (2009). Regulatory T cells are key cerebroprotective immunomodulators in acute experimental stroke. Nature Medicine, 15, 192–199. 10.1038/nm.1927 19169263

[phy216118-bib-0017] Lyden, P. D. , Bosetti, F. , Diniz, M. A. , Rogatko, A. , Koenig, J. I. , Lamb, J. , Nagarkatti, K. A. , Cabeen, R. P. , Hess, D. C. , Kamat, P. K. , Khan, M. B. , Wood, K. , Dhandapani, K. , Arbab, A. S. , Leira, E. C. , Chauhan, A. K. , Dhanesha, N. , Patel, R. B. , Kumskova, M. , … Whittier, J. P. W. (2022). The stroke preclinical assessment network: Rationale, design, feasibility, and stage 1 results. Stroke, 53, 1802–1812. 10.1161/strokeaha.121.038047 35354299 PMC9038686

[phy216118-bib-0018] Lyden, P. D. , Diniz, M. A. , Bosetti, F. , Lamb, J. , Nagarkatti, K. A. , Rogatko, A. , Kim, S. , Cabeen, R. P. , Koenig, J. I. , Akhter, K. , Arbab, A. S. , Avery, B. D. , Beatty, H. E. , Bibic, A. , Cao, S. , Simoes Braga Boisserand, L. , Chamorro, A. , Chauhan, A. , Diaz‐Perez, S. , … Sansing, L. H. (2023). A multi‐laboratory preclinical trial in rodents to assess treatment candidates for acute ischemic stroke. Science Translational Medicine, 15, eadg8656. 10.1126/scitranslmed.adg8656 37729432

[phy216118-bib-0019] Macrae, I. M. (2011). Preclinical stroke research–advantages and disadvantages of the most common rodent models of focal ischaemia. British Journal of Pharmacology, 164, 1062–1078. 10.1111/j.1476-5381.2011.01398.x 21457227 PMC3229752

[phy216118-bib-0020] Mandeville, E. T. , Ayata, C. , Zheng, Y. , & Mandeville, J. B. (2017). Translational MR neuroimaging of stroke and recovery. Translational Stroke Research, 8, 22–32. 10.1007/s12975-016-0497-z 27578048 PMC5243530

[phy216118-bib-0021] McKeown, M. E. , Prasad, A. , Kobsa, J. , Top, I. , Snider, S. B. , Kidwell, C. , Campbell, B. C. V. , Davis, S. M. , Donnan, G. A. , Lev, M. , Sheth, K. N. , Petersen, N. , Kimberly, W. T. , & Bevers, M. B. (2022). Midline shift greater than 3 mm independently predicts outcome after ischemic stroke. Neurocritical Care, 36, 46–51. 10.1007/s12028-021-01341-x 34494212 PMC8813904

[phy216118-bib-0022] Mingrone, A. , & Kaffman, A. (2020). The promise of automated home‐cage monitoring in improving translational utility of psychiatric research in rodents. Frontiers in Neuroscience, 14, 618593. 10.3389/fnins.2020.618593 33390898 PMC7773806

[phy216118-bib-0023] Morrison, H. , Young, K. , Qureshi, M. , Rowe, R. K. , & Lifshitz, J. (2017). Quantitative microglia analyses reveal diverse morphologic responses in the rat cortex after diffuse brain injury. Scientific Reports, 7, 13211. 10.1038/s41598-017-13581-z 29038483 PMC5643511

[phy216118-bib-0024] Morrison, H. W. , & Filosa, J. (2013). A quantitative spatiotemporal analysis of microglia morphology during ischemic stroke and reperfusion. Journal of Neuroinflammation, 10, 4. 23311642 10.1186/1742-2094-10-4 PMC3570327

[phy216118-bib-0025] Morrison, H. W. , & Filosa, J. A. (2016). Sex differences in astrocyte and microglia responses immediately following middle cerebral artery occlusion in adult mice. Neuroscience, 339, 85–99. 10.1016/j.neuroscience.2016.09.047 27717807 PMC5118180

[phy216118-bib-0026] Morrison, H. W. , & Filosa, J. A. (2019). Stroke and the neurovascular unit: Glial cells, sex differences, and hypertension. American Journal of Physiology. Cell Physiology, 316, C325–c339. 10.1152/ajpcell.00333.2018 30601672 PMC6457101

[phy216118-bib-0027] Nguyen, T. V. , Frye, J. B. , Zbesko, J. C. , Stepanovic, K. , Hayes, M. , Urzua, A. , Serrano, G. , Beach, T. G. , & Doyle, K. P. (2016). Multiplex immunoassay characterization and species comparison of inflammation in acute and non‐acute ischemic infarcts in human and mouse brain tissue. Acta Neuropathologica Communications, 4, 100. 10.1186/s40478-016-0371-y 27600707 PMC5011964

[phy216118-bib-0028] Nielsen, H. H. , Soares, C. B. , Høgedal, S. S. , Madsen, J. S. , Hansen, R. B. , Christensen, A. A. , Madsen, C. , Clausen, B. H. , Frich, L. H. , Degn, M. , Sibbersen, C. , & Lambertsen, K. L. (2020). Acute neurofilament light chain plasma levels correlate with stroke severity and clinical outcome in ischemic stroke patients. Frontiers in Neurology, 11, 448. 10.3389/fneur.2020.00448 32595585 PMC7300211

[phy216118-bib-0029] O'Collins, V. E. , Macleod, M. R. , Donnan, G. A. , Horky, L. L. , van der Worp, B. H. , & Howells, D. W. (2006). 1,026 experimental treatments in acute stroke. Annals of Neurology, 59, 467–477. 10.1002/ana.20741 16453316

[phy216118-bib-0030] Osa García, A. , Brambati, S. M. , Desautels, A. , & Marcotte, K. (2022). Timing stroke: A review on stroke pathophysiology and its influence over time on diffusion measures. Journal of the Neurological Sciences, 441, 120377. 10.1016/j.jns.2022.120377 36049374

[phy216118-bib-0031] Percie du Sert, N. , Hurst, V. , Ahluwalia, A. , Alam, S. , Avey, M. T. , Baker, M. , Browne, W. J. , Clark, A. , Cuthill, I. C. , Dirnagl, U. , Emerson, M. , Garner, P. , Holgate, S. T. , Howells, D. W. , Karp, N. A. , Lazic, S. E. , Lidster, K. , MacCallum, C. J. , Macleod, M. , … Würbel, H. (2020). The ARRIVE guidelines 2.0: Updated guidelines for reporting animal research. The Journal of Physiology, 598, 3793–3801. 10.1113/jp280389 32666574 PMC7610696

[phy216118-bib-0032] Pierpaoli, L. , Walker, M. O. I. A. , Barnett, P. , Basser, L.‐C. , Chang, C. , Koay, S. , Pajevic, G. R. , Sarlls, J. , & Wu, M. (2019). Stockholm, Sweden, abstract #1597 (ISMRM 18th annual meeting, Stockholm, Sweden, abstract #15972019).

[phy216118-bib-0033] Schuchman, E. H. , & Desnick, R. J. (2017). Types a and B Niemann‐pick disease. Molecular Genetics and Metabolism, 120, 27–33. 10.1016/j.ymgme.2016.12.008 28164782 PMC5347465

[phy216118-bib-0034] Sierra, A. , Abiega, O. , Shahraz, A. , & Neumann, H. (2013). Janus‐faced microglia: Beneficial and detrimental consequences of microglial phagocytosis. Frontiers in Cellular Neuroscience, 7, 6. 10.3389/fncel.2013.00006 23386811 PMC3558702

[phy216118-bib-0035] Sofroniew, M. V. (2009). Molecular dissection of reactive astrogliosis and glial scar formation. Trends in Neurosciences, 32, 638–647. 10.1016/j.tins.2009.08.002 19782411 PMC2787735

[phy216118-bib-0036] Thayabaranathan, T. , Kim, J. , Cadilhac, D. A. , Thrift, A. G. , Donnan, G. A. , Howard, G. , Howard, V. J. , Rothwell, P. M. , Feigin, V. , Norrving, B. , Owolabi, M. , Pandian, J. , Liu, L. , & Olaiya, M. T. (2022). Global stroke statistics 2022. International Journal of Stroke, 17, 946–956. 10.1177/17474930221123175 35975986 PMC9980380

[phy216118-bib-0037] Tiedt, S. , Duering, M. , Barro, C. , Kaya, A. G. , Boeck, J. , Bode, F. J. , Klein, M. , Dorn, F. , Gesierich, B. , Kellert, L. , Ertl‐Wagner, B. , Goertler, M. W. , Petzold, G. C. , Kuhle, J. , Wollenweber, F. A. , Peters, N. , & Dichgans, M. (2018). Serum neurofilament light: A biomarker of neuroaxonal injury after ischemic stroke. Neurology, 91, e1338–e1347. 10.1212/wnl.0000000000006282 30217937

[phy216118-bib-0038] Tournier, J. D. , Smith, R. , Raffelt, D. , Tabbara, R. , Dhollander, T. , Pietsch, M. , Christiaens, D. , Jeurissen, B. , Yeh, C. H. , & Connelly, A. (2019). MRtrix3: A fast, flexible and open software framework for medical image processing and visualisation. NeuroImage, 202, 116137. 10.1016/j.neuroimage.2019.116137 31473352

[phy216118-bib-0039] Tremblay, M. E. , Stevens, B. , Sierra, A. , Wake, H. , Bessis, A. , & Nimmerjahn, A. (2011). The role of microglia in the healthy brain. The Journal of Neuroscience, 31, 16064–16069. 22072657 10.1523/JNEUROSCI.4158-11.2011 PMC6633221

[phy216118-bib-0040] Tsao, C. W. , Aday, A. W. , Almarzooq, Z. I. , Anderson, C. A. M. , Arora, P. , Avery, C. L. , Baker‐Smith, C. M. , Beaton, A. Z. , Boehme, A. K. , Buxton, A. E. , Commodore‐Mensah, Y. , Elkind, M. S. V. , Evenson, K. R. , Eze‐Nliam, C. , Fugar, S. , Generoso, G. , Heard, D. G. , Hiremath, S. , Ho, J. E. , … *American Heart Association Council on Epidemiology and Prevention Statistics Committee and Stroke Statistics Subcommittee* . (2023). Heart disease and stroke statistics‐2023 update: A report from the American Heart Association. Circulation, 147, e93–e621. 10.1161/cir.0000000000001123 36695182 PMC12135016

[phy216118-bib-0041] Vindegaard, N. , Muñoz‐Briones, C. , el Ali, H. H. , Kristensen, L. K. , Rasmussen, R. S. , Johansen, F. F. , & Hasseldam, H. (2017). T‐cells and macrophages peak weeks after experimental stroke: Spatial and temporal characteristics. Neuropathology, 37, 407–414. 10.1111/neup.12387 28517732

[phy216118-bib-0042] Yoo, A. J. , Sheth, K. N. , Kimberly, W. T. , Chaudhry, Z. A. , Elm, J. J. , Jacobson, S. , Davis, S. M. , Donnan, G. A. , Albers, G. W. , Stern, B. J. , & González, R. G. (2013). Validating imaging biomarkers of cerebral edema in patients with severe ischemic stroke. Journal of Stroke and Cerebrovascular Diseases, 22, 742–749. 10.1016/j.jstrokecerebrovasdis.2012.01.002 22325573 PMC3529850

[phy216118-bib-0043] Young, K. , & Morrison, H. (2018). Quantifying microglia morphology from photomicrographs of immunohistochemistry prepared tissue using ImageJ. Journal of Visualized Experiments, 136, 57648. 10.3791/57648 PMC6103256 29939190

[phy216118-bib-0044] Young, K. F. , Gardner, R. , Sariana, V. , Whitman, S. A. , Bartlett, M. J. , Falk, T. , & Morrison, H. W. (2021). Can quantifying morphology and TMEM119 expression distinguish between microglia and infiltrating macrophages after ischemic stroke and reperfusion in male and female mice? Journal of Neuroinflammation, 18, 58. 10.1186/s12974-021-02105-2 33618737 PMC7901206

[phy216118-bib-0045] Zbesko, J. C. , Frye, J. B. , Becktel, D. A. , Gerardo, D. K. , Stokes, J. , Calderon, K. , Nguyen, T. V. V. , Bhattacharya, D. , & Doyle, K. P. (2020). IgA natural antibodies are produced following T‐cell independent B‐cell activation following stroke. Brain, Behavior, and Immunity, 91, 578–586. 10.1016/j.bbi.2020.09.014 32956832 PMC8279117

[phy216118-bib-0046] Zbesko, J. C. , Nguyen, T. V. V. , Yang, T. , Frye, J. B. , Hussain, O. , Hayes, M. , Chung, A. , Day, W. A. , Stepanovic, K. , Krumberger, M. , Mona, J. , Longo, F. M. , & Doyle, K. P. (2018). Glial scars are permeable to the neurotoxic environment of chronic stroke infarcts. Neurobiology of Disease, 112, 63–78. 10.1016/j.nbd.2018.01.007 29331263 PMC5851450

[phy216118-bib-0047] Zbesko, J. C. , Stokes, J. , Becktel, D. A. , & Doyle, K. P. (2023). Targeting foam cell formation to improve recovery from ischemic stroke. Neurobiology of Disease, 181, 106130. 10.1016/j.nbd.2023.106130 37068641 PMC10993857

